# Gout in a 15-year-old boy with juvenile idiopathic arthritis: a case study

**DOI:** 10.1186/1546-0096-12-1

**Published:** 2014-01-06

**Authors:** Hallie Morris, Kristen Grant, Geetika Khanna, Andrew J White

**Affiliations:** 1 Boston Combined Residency Program, PGY1, Boston, Mass, USA; 2 Washington University School of Medicine, WUSM IV, St Louis, Missouri, USA; 3 Division of Radiology, Washington University School of Medicine, St Louis, Missouri, USA; 4 Division of Pediatric Rheumatology, Washington University School of Medicine, St Louis, Missouri, USA

**Keywords:** Juvenile idiopathic arthritis, Gout, Obesity, Ankle pain, Adolescent, Joint pain, Autoimmune, Osteochondritis dissecans, Treatment failure

## Abstract

Joint pain is a common complaint in pediatrics and is most often attributed to overuse or injury. In the face of persistent, severe, or recurrent symptoms, the differential typically expands to include bony or structural causes versus rheumatologic conditions. Rarely, a child has two distinct causes for joint pain. In this case, an obese 15-year-old male was diagnosed with gout, a disease common in adults but virtually ignored in the field of pediatrics. The presence of juvenile idiopathic arthritis (JIA) complicated and delayed the consideration of this second diagnosis. Indeed, the absence of gout from this patient’s differential diagnosis resulted in a greater than two-year delay in receiving treatment. The patients’ BMI was 47.4, and he was also mis-diagnosed with osteochondritis dissecans and underwent medical treatment for JIA, assorted imaging studies, and multiple surgical procedures before the key history of increased pain with red meat ingestion, noticed by the patient, and a subsequent elevated uric acid confirmed his ultimate diagnosis. With the increased prevalence of obesity in the adolescent population, the diagnosis of gout should be an important consideration in the differential diagnosis for an arthritic joint in an overweight patient, regardless of age.

## Background

In the pediatric population, there are numerous causes of joint pain, stiffness, and swelling. Many can be attributed to minor activity or overuse-related injury, especially in the overweight and obese populations [1], but in the face of persistent, severe, or recurrent symptoms, other diagnoses must be considered. These typically fall into two categories in children and adolescents: bony or structural causes [2], or rheumatologic conditions [3]. Careful history and physical examination, along with use of imaging and laboratory studies, can often distinguish between the two [4,5]; however, when a complete work-up is performed and no clear answer emerges, the differential must be expanded [6]. In the rare case in which a firm primary diagnosis has been made, it is more difficult still to consider additional, secondary, causes of joint pain.

The following case describes an adolescent young man with severe ankle pain, as well as multiple other joint complaints, who was correctly diagnosed and treated for polyarticular juvenile idiopathic arthritis. While the majority of his joints improved, his ankle continued to be extremely tender and swollen. After two years of aggressive medical treatment, surgical procedures, and multiple imaging studies, it was careful probing into his history and classic physical examination findings that ultimately led to the additional diagnosis of gout.

## Case presentation

A 15-year-old obese Hispanic young man presented to the orthopedic surgery service with right-sided ankle pain. His pain began after a sports injury approximately one year prior to presentation but did not respond to immobilization, physical therapy, or prolonged rest. The pain was located at the medial side of the ankle, was worse with activity, and was accompanied by intermittent swelling. He took ibuprofen at night occasionally that provided modest relief.

His past medical history was unremarkable. Family history was negative for autoimmune disease including JIA. His only medication was occasional ibuprofen, and his immunizations were up to date.

On examination, the patient was heavyset, weighing 142.8 kg and standing 173.6 cm tall, with a BMI of 47.4. His vital signs were normal and he was in no distress. Musculoskeletal exam revealed bilateral limited ankle dorsiflexion, worse on the right. He had tenderness to palpation over the medial aspect of his ankle just anterior to the medial malleolus. All other joints were normal.

His initial work-up consisted of radiographs of his right ankle, showing evidence of a healing osteochondritis dissecans (OCD) lesion. He was instructed to continue physical therapy and follow up in three months with the possibility of arthroscopy of the affected joint if there was no improvement.

At follow-up, his pain was unchanged. Radiographs showed the previously seen presumed OCD lesion, now with a sclerotic border. He was scheduled for an MRI of the ankle, which was read as more consistent with a chondroblastoma versus an inflammatory process.

Several months later, he began complaining of left shoulder pain and decreased range of motion of gradual onset. He denied any trauma causing this new complaint. He was again seen by the orthopedic service and found to have significantly decreased range of motion and AC joint tenderness. Radiographs of the shoulder did not show any abnormalities. He was diagnosed with adhesive capsulitis and instructed to perform stretching exercises and take acetaminophen for pain. An MRI of the shoulder was performed given the unusual age of presentation for adhesive capsulitis, which revealed evidence of inflammation and synovitis consistent with juvenile idiopathic arthritis. He was referred to the rheumatology service.

The patient described daily pain and some morning stiffness for several weeks at a time, which would then subside for several weeks before returning. The pain and stiffness involved the right elbow, left knee, right wrist and several fingers in addition to the left shoulder and right ankle previously described. Examination by the pediatric rheumatologist noted improvement of his range of motion of the left shoulder with his home exercises and was back within normal limits. He did have moderate pain with extension of the shoulder. His left wrist was also tender with limited range of motion. His range of motion was limited bilaterally in the ankles and multiple PIP joints were painful and swollen. Considering the symmetric joint distribution, involvement of PIPs, and an elevated rheumatoid factor of 24.7 IV/mL (nl 0.0-13.9), the patient’s presentation was felt to be most consistent with early onset rheumatoid arthritis, or RF + polyarticular JIA. Subsequently, an anti-CCP antibody test was positive. He was prescribed naproxen and methotrexate. Laboratory studies from this initial visit were also notable for an elevated CRP 6.7 mg/dL, WBC 12.6 K/uL, Hgb of 12.3 g/dL, and ESR of 61 mm/hr. ANA, dsDNA, and HLA-B27 were negative.

The patient presented for follow-up approximately 2.5 months after beginning the naproxen and methotrexate regimen. At that time, the pain in the majority of his joints had improved substantially, however, the right ankle had become more tender and swollen. The pain was worse in the morning, to the point that he began using crutches. He was started on twice weekly etanercept injections, but when he experienced no relief, was subsequently switched to adalimumab and then to rituximab.

**Figure 1:**
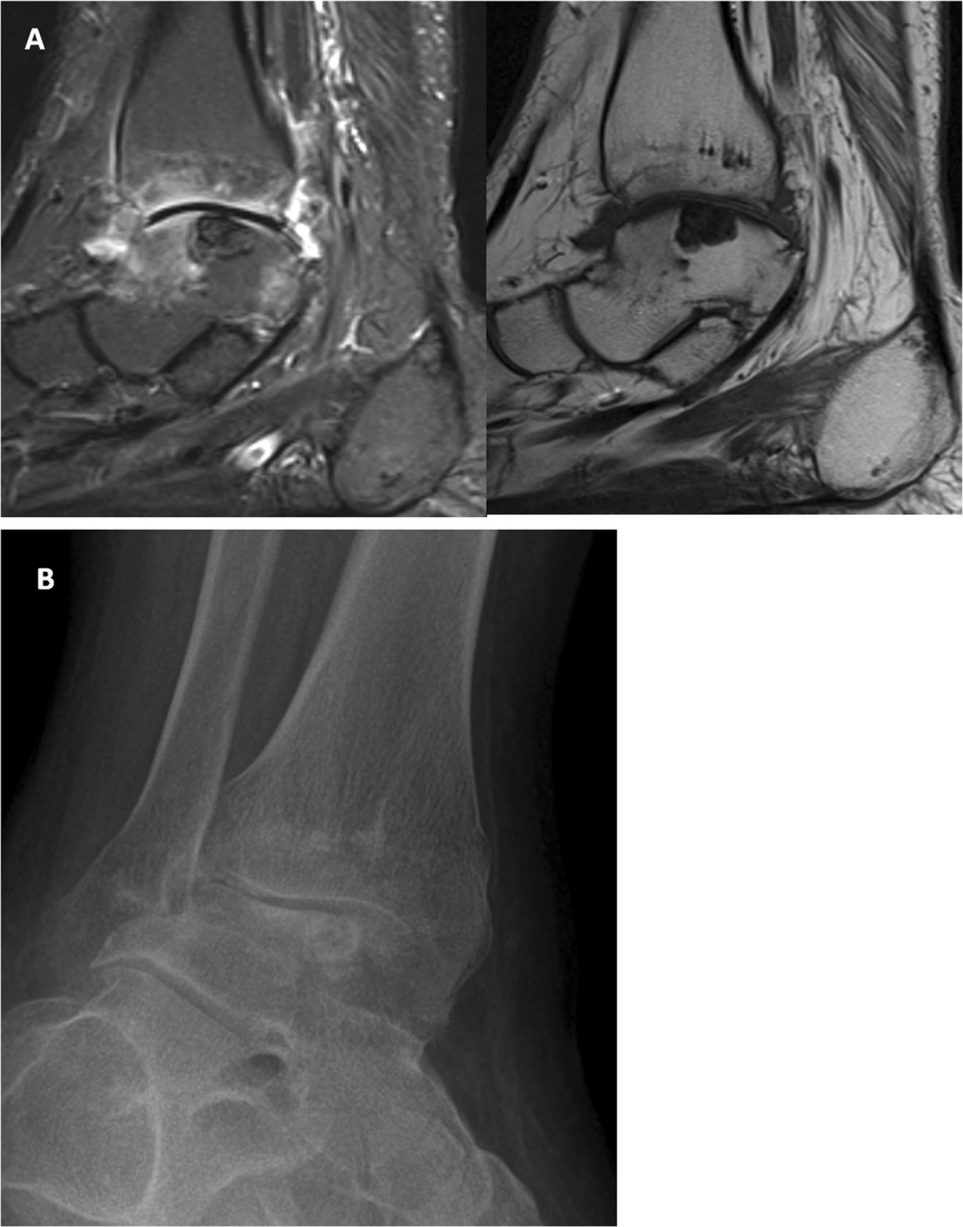
**Imaging of gouty joint in an adolescent. (A)** Synovitis on MRI with and without IV contrast of the right ankle with osteitis in the distal tibia and talar dome. Talar dome lesion was thought to represent an osteochondritis dissecans/osteonecrosis. **(B)** Radiographs performed 15 months later show marked joint space loss with persistent talar dome lesion which likely represents an intraosseous tophus.

Given the lack of response of the patient’s ankle to this therapeutic regimen (Figure 1) over the course of the next year, despite improvement in his other joints, arthroscopic exploration of the right ankle with synovectomy and potential OCD curettage was performed. During surgery, the patient was found to have “significant debris” including a “crystalline white substance” within the joint space, which was attributed by the surgeons to “the previous steroid injection.” The fluid was not sent for culture, cell count or crystal studies, despite the fact that the patient had not ever received a steroid injection in that joint. The debris was removed, but not sent for pathology.

After recovery from surgery, the patient returned to the rheumatology service for further management of his JIA. He continued to have pain and stiffness of his right ankle as well as several other joints that was difficult to manage. He was eventually started on a low dose of prednisone, previously avoided given his weight, which did offer moderate symptomatic relief. Ultimately the medication regimen of prednisone, etanercept, methotrexate, naproxen, and sulfasalazine had the greatest impact on his JIA symptoms, although his right ankle continued to be the most painful joint.

**Figure 2:**
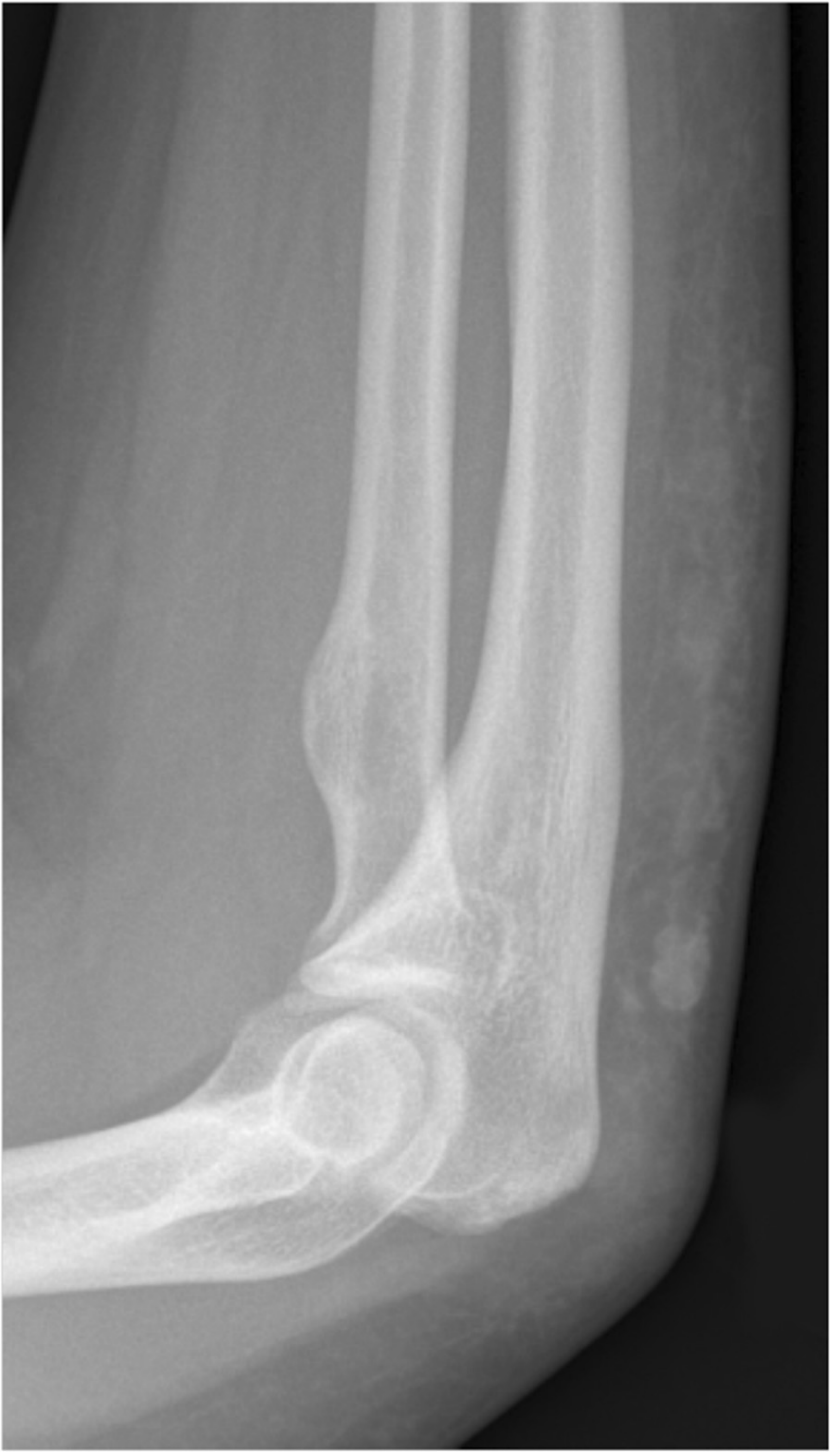
**X-ray imaging of gouty tophus in an adolescent.** Lateral view of the forearm shows subcutaneous nodules along the dorsal aspect of the proximal forearm.

2.5 years after he first presented, the patient developed multiple non-tender scattered subcutaneous nodules over the extensor surface of his bilateral forearms, his left elbow, and his right knee (Figure 2). Their etiology was unclear, but attributed to rheumatoid nodules. Four months later, however, the patient spontaneously noted that his ankle pain seemed to worsen following the ingestion of red meat. With this new data in hand, a uric acid level was sent and was extremely elevated at 13.3 mg/dL. The nodules were deemed to be consistent with tophi, virtually pathognomonic for gout. The patient was started on colchicine and then allopurinol and improved. Over the course of time he did begin to complain of pain in his big toe, a more classic presentation of gout. Uric acid levels however, remained high, running between 11.7 and 13.5 mg/dL over the following year.

At the time of the patient’s transition to adult care, he continued to exhibit active symptomatology, with joint pain in the knees, elbows, and ankles, limited range of motion in the aforementioned joints as well as his wrists, MCP’s and PIP’s, and difficulty ambulating. He continues to be treated for both JIA and gout.

## Discussion

Joint pain is a common complaint in pediatrics and in the face of persistent, severe, or recurrent symptoms, the differential typically expands to include bony or structural causes versus rheumatologic conditions. In this case, however, diagnosis was complicated because the child had two distinct etiologies causing joint pain. Moreover, the patient’s second diagnosis was gout, a very uncommon condition in a pediatric patient, even in the setting of morbid obesity.

Poly-articular juvenile idiopathic arthritis was considered as the unifying diagnosis when the patient presented with involvement of multiple joints. The typical presenting features of JIA are morning stiffness, pain and swelling of the joints, limited range of motion, and joint contractures [7]. Radiographs may show some soft tissue swelling and osteopenia early, with subchondral sclerosis and erosions evident after long-standing disease, but MRI has been shown to be more sensitive for bone marrow edema and tenosynovitis, as well as bone erosions, cartilage lesions, and synovial hypertrophy [8]. JIA is most often seen in children of European descent, but affects children throughout the world. The disease is thought to be idiopathic with its cause poorly understood, although there is mounting evidence that autoimmunity may be involved [7]. There is also thought to be potential genetic susceptibility in affected individuals [9]. While the majority of the patient’s overall complaints did seem to fit the diagnosis of JIA both in his clinical presentation and laboratory and imaging results, as well as his response to treatment, his right ankle continued to be refractory to treatment.

The acquisition of additional history led to the consideration of gout as a second diagnosis. Historically, gout has affected predominantly older, overweight men but in more recent years the male to female ratio has fallen to 2:1 [10]. A resurgence of gout across the population has been noted in recent years, and juvenile gout has also begun to be reported, with many of the cases being due solely to known risk factors such as being overweight [11,12]. On imaging, plain films may show little evidence of gout in early stages, but later in the course can show joint effusions, bony erosions, or tophi within the joint. Gout can appear similar to other arthritides on MRI, with mild bone marrow edema, tenosynovitis, and bony erosions, making diagnosis difficult but important to consider, especially in the setting of an overweight or obese patient. However, if tophi are present, MRI is able to detect this as a potentially differentiating characteristic. Other imaging studies may be of more use to differentiate gout from other diagnoses, as ultrasound may be able to detect crystals within the joint space, and can even differentiate between gout and pseudogout [13]. The gold standard for diagnosing gout remains the acquisition of urate crystals from synovial fluid. In the absence of this data, however, the presence of 2 of the 3 Rome clinical criteria (uric acid >7.0 mg/dL, history of painful joint with abrupt onset and remission within 2 weeks, and presence of tophus), as were existent in our patient, was found to have a positive predictive value of 76.9%, and a specificity of 88.5% [14].

While it seems likely the patient did have gout, gout alone does not seem most likely given the clinical picture. Gout simulating JIA is a possibility; a patient with untreated gout can develop bony erosions and deformities, leading to the disappearance of the intercritical periods which are usually pathognomonic of gout. Typically, however, one would expect gout to present as an episodic arthritis. Even in untreated individuals, complete resolution of the earliest attacks nearly always occurs within several weeks, which this patient never experienced. Moreover, the symmetric joint distribution with involvement of the PIPs along with a positive rheumatoid factor and CCP antibody point toward the concurrent JIA diagnosis in this case.

There were several missed opportunities to diagnose this patient earlier in his course. First, gout was not considered as a part of the original differential because of its propensity to affect older individuals. As the epidemic of childhood obesity grows, adult conditions usually a result of long term lifestyle consequences are being seen more frequently in the pediatric population, most notably type II diabetes but also musculoskeletal complaints and, as in this case, gout.

Secondly, if gout had been on the differential, the radiologic features may have been recognized as being consistent with this condition. Third, a uric acid level was not sent until after the additional diet history was obtained. And finally, proper examination of joint fluid, from either aspiration or surgical debridement, would have revealed the presence of negatively birefringent crystals, providing a timely diagnosis.

## Conclusion

In conclusion, gout was diagnosed in this teenage patient with longstanding juvenile idiopathic arthritis. The diagnosis of gout should therefore be an important element of the differential for a refractory painful joint in an overweight patient regardless of age, and regardless of pre-existing diagnoses. Failing to consider this diagnosis may result in delay of optimal treatment and cause long-term effects of bone erosion and joint destruction. Sending joint fluid for crystalline analysis, checking uric acid levels, and performing imaging studies, specifically non-invasive, cost effective modalities such as ultrasound, are all reasonable parts of a complete work-up in any child with arthritis.

## Consent

Written informed consent was obtained from the patient for publication of this Case Report and any accompanying images. A copy of the written consent is available for review by the Editor-in-Chief of this journal.

## Competing interests

The authors declare that they have no competing interests.

## Authors’ contributions

KG participated in background research and drafting of the manuscript. HM was involved in drafting and revision of the manuscript. GK contributed figures and their impressions. AW conceived of the study, participated in its coordination, and was involved in drafting and revision of the manuscript. All authors read and approved of the final manuscript.
